# Prevalence of alcohol-related pathologies at autopsy: Estonian Forensic Study of Alcohol and Premature Death

**DOI:** 10.1111/add.12695

**Published:** 2014-08-28

**Authors:** Jana Tuusov, Katrin Lang, Marika Väli, Kersti Pärna, Mailis Tõnisson, Inge Ringmets, Martin McKee, Anders Helander, David A Leon

**Affiliations:** 1 Institute of Pathological Anatomy and Forensic Medicine, University of TartuTartu, Estonia; 2 Estonian Forensic Science InstituteTallinn, Estonia; 3 Department of Public Health, University of TartuTartu, Estonia; 4 Internal Medicine Clinic, University of TartuTartu, Estonia; 5 London School of Hygiene and Tropical MedicineLondon, UK; 6 Department of Laboratory Medicine, Karolinska Institutet and Karolinska University LaboratoryStockholm, Sweden; 7 The Arctic University of NorwayTromsø, Norway

**Keywords:** Alcohol drinking, alcohol-related pathologies, epidemiology, Estonia/forensic autopsy, ICD codes, post-mortem alcohol biomarkers.

## Abstract

**Aims:**

Alcohol can induce diverse serious pathologies, yet this complexity may be obscured when alcohol-related deaths are classified according to a single underlying cause. We sought to quantify this issue and its implications for analysing mortality data.

**Design, Setting and Participants:**

Cross-sectional study included 554 men aged 25–54 in Estonia undergoing forensic autopsy in 2008–09.

**Measurements:**

Potentially alcohol-related pathologies were identified following macroscopic and histological examination. Alcohol biomarkers levels were determined. For a subset (26%), drinking behaviour was provided by next-of-kin. The Estonian Statistics Office provided underlying cause of death.

**Findings:**

Most deaths (75%) showed evidence of potentially alcohol-related pathologies, and 32% had pathologies in two or more organs. The liver was most commonly affected [60.5%, 95% confidence interval (CI) = 56.3–64.6] followed by the lungs (18.6%, 95% CI = 15.4–22.1), stomach (17.5%, 95% CI = 14.4–20.9), pancreas (14.1%, 95% CI = 11.3–17.3), heart (4.9%, 95% CI = 3.2–7.0) and oesophagus (1.4%, 95% CI = 0.6–2.8). Only a minority with liver pathology had a second pathology. The number of pathologies correlated with alcohol biomarkers (phosphatidylethanol, gamma-glytamyl transpeptidase in blood, ethylglucuronide, ethylsulphate in urine). Despite the high prevalence of liver pathology, few deaths had alcoholic liver disease specified as the underlying cause.

**Conclusion:**

The majority of 554 men aged 25–54 undergoing forensic autopsy in Estonia in 2008–09 showed evidence of alcohol-related pathology. However, the recording of deaths by underlying cause failed to capture the scale and nature of alcohol-induced pathologies found.

## Introduction

Alcohol can cause or be implicated in death in many ways, both through acute direct (e.g. alcohol poisoning) and indirect (injuries and violence) pathways 1, and as a result of chronic effects on organs such as the digestive and cardiovascular systems 2–6. Within each organ system, there is a spectrum of possible alcohol-related pathological processes. In the digestive system, the liver may exhibit steatosis (fatty liver), steatohepatitis (alcoholic hepatitis) or cirrhosis 7,8. Damage may also occur in the upper gastrointestinal tract 9 in the form of gastritis, as well as oesophageal varices in those with portal hypertension secondary to cirrhosis.

The association between alcohol and cardiovascular disease is more contentious, with evidence that moderate consumption may be protective in those at risk of atheromatous disease, while there is emerging evidence that heavy irregular drinking increases the risk of mortality from heart disease 10. In addition, heavy drinking is associated with hypertension and stroke 11, and can lead to cardiomyopathy. Alcohol may also cause damage to other organ systems, such as the respiratory system, either by direct injury following aspiration of gastric contents or as a consequence of reduced immune function predisposing heavy drinkers to diseases such as pneumonia and tuberculosis 12,13, and to the nervous system, whether due to associated thiamine deficiency or by predisposing individuals to dementia 14.

Given the range of alcohol-related pathologies, there is inevitably a loss of information and potential misclassification when assigning a single underlying cause of death. This poses a challenge, both for those certifying the cause of death (e.g. pathologists) and for epidemiologists and others attempting to interpret these data 15. Although these issues are widely acknowledged, we have been unable to find any systematic quantification of the spectrum of alcohol-related pathologies found at autopsy, and how this relates to the statistical codes selected to capture the single underlying cause of death, even though this must be an issue in all countries. In this paper we address this gap, using the results of a forensic autopsy study conducted in Estonia in 2008–09.

## Methods

### Study population

The study used data from the Estonian Forensic Study of Alcohol and Premature Death, details of which have been reported elsewhere 16. In brief, the target study population was men dying at ages 25–54 years in Estonia subject to forensic autopsy in 2008–09 (*n* = 1299). The study is based on 595 (46%) of these autopsies. Exclusions were a consequence of pressure of routine work in the forensic centres. Characteristics of eligible deaths included and excluded from the study showed no significant differences with regard to age group, but there were slightly more deaths from external causes and with high blood alcohol concentrations among those included, as reported elsewhere 16.

### Autopsy procedures

At autopsy, a systematic examination of the organs was undertaken according to a standardized research protocol developed following a literature review and consultation with forensic pathologists. The results were recorded by the forensic specialist using a structured proforma. For this paper we restricted our attention to the 554 (93%) autopsies which had complete information about the presence or absence of pathology in each organ system considered. In the 41 excluded cases, the status of some or all organs could not be determined due to putrefaction, major trauma or fire.

Tissue sections were taken for histological examination from the parenchyma of both lungs, liver, spleen, pancreas, heart, brain, stomach and both kidneys. These were fixed immediately in buffered 10% formalin (pH 7.4) for 24 hours and embedded in paraffin wax. As described elsewhere 16, liver enzymes (aspartate aminotransferase, alanine aminotransferase and gamma-glutamyltransferase in serum) were assayed and ethanol concentrations in blood, urine and vitreous humour were measured, as were direct alcohol biomarkers (i.e. ethanol metabolites) phosphatidylethanol (PEth) in whole blood and ethyl glucuronide (EtG) and ethyl sulphate (EtS) in urine.

We defined six classes of potentially alcohol-related pathologies of the liver, pancreas, lung, stomach, oesophagus and heart (Table 1). The alcohol-related pathologies were determined from a combination of gross examination of organs and histology. The presence of oesophageal varices was determined macroscopically. The presence or absence of each class of pathology was coded (by J.T.) as a binary variable, with no intermediate gradations.

**Table 1:** Criteria for determining presence or absence of each class of potentially alcohol-related pathology and autopsies with positive findings for various classes of potentially alcohol-related pathologies (number and percentage) from autopsies with known data on all cases (*n* = 554)

Organ	Type of potentially alcohol-related pathology	Positive finding	
		n	% (95% CI)
Liver	Focal/diffuse steatosis, complete/incomplete fibrosis, complete/incomplete cirrhosis	335	60.5 (56.3–64.6)
Pancreas	Acute and chronic pancreatitis	78	14.1 (11.3–17.3)
Lung	Pneumonia and/or aspiration of gastric content	103	18.6 (15.4–22.1)
Stomach	Gastritis	97	17.5 (14.4–20.9)
Oesophagus	Varices (determined macroscopically)	8	1.4 (0.6–2.8)
Heart	Dilative (and alcoholic) cardiomyopathy	27	4.9 (3.2–7.0)

CI = confidence interval.

### Data on deceased

For a subset of cases (144 of 554; 26%) who had lived in one of the five major towns of Estonia, we successfully interviewed the next-of-kin about the drinking behaviour of the deceased. The survey instrument was adapted from the one developed and validated in our earlier research using proxy informants in Russia 17.

We obtained the underlying cause of death for each subject, as assigned and coded by the Estonian Death Registry. In the analysis of these data, we focused on the following underlying causes: alcohol dependence syndrome (ICD-10 F10.2), alcoholic cardiomyopathy (I42.6), alcoholic liver disease (K70) and acute alcohol poisoning (X45), cardiovascular diseases (I00–I99, except I42), other cardiomyopathies (I42, except I42.6), digestive diseases (K00–K93, except K70), respiratory diseases (J00–J99) and external causes (V01–Y98, except X45). All remaining underlying causes were assembled into a single aggregate category (‘other’).

### Statistical analysis

Frequency tables and cross-tabulations were examined, with means, standard deviation and medians of biomarkers as appropriate. The association between biomarker levels and each class of pathology was assessed using linear regression with log transformed biomarker values. The non-parametric Cuzick test for trend across ordered groups was used for identifying the relationship between frequency of drinking and pathology classes among drinkers. Logistic regression was used to estimate the strength of association between cause of death and the number of positive pathology classes adjusted for age. For this analysis, cause of death was dichotomized into alcohol-related (I42.6, K70, F10.2, X45) and other (all other) causes. Data were analysed using Stata version 11 18.

## Results

Table 1 shows the numbers and proportions of autopsies with each form of potentially alcohol-related pathology. The most common pathology was that affecting the liver, with 60% of deaths showing evidence of steatosis, fibrosis or cirrhosis. Pancreatic, lung or gastric pathology was detected in between 14 and 19% of all cases. Evidence of damage to the heart in the form of cardiomyopathy was much less frequent (5%), and oesophageal varices were found only rarely.

Table 2 shows the distribution of cases according to the number of classes of pathology, for the total sample and for two age groups. Overall, 75% of the subjects showed evidence of one or more classes of pathology, and 32% had evidence of two or more classes. The prevalence of two or more pathologies was significantly higher among the older (45–54 years) men (*P* = 0.002, Fisher's exact test).

**Table 2:** Distribution of autopsies according to the number of potentially alcohol-related pathologies found by age

Number of classes of pathology	Age in years				Total	
		25–44		45–54		
	n	% (CI 95%)	n	% (CI 95%)	n	% (CI 95%)
0	73	36.9 (30.1–44.0)	68	19.1 (15.1–23.6)	141	25.5 (21.9–29.3)
1	77	38.9 (32.1–46.1)	157	44.1 (38.9–49.4)	234	42.4 (38.1–46.5)
2	39	19.7 (14.4–25.0)	92	25.8 (21.4–30.7)	131	23.7 (20.2–27.4)
3+	9	4.5 (2.1–8.5)	39	11.0 (7.9–14.7)	48	8.7 (6.5–11.3)
Total	198	100	356	100	554	100

CI = confidence interval.

Table 3 shows the relationship between levels of the various biomarkers of recent (ethanol, EtG, EtS) or heavy (PEth, GGT) drinking and the number of positive pathology classes. No associations were seen with the mean ethanol concentration in blood, urine or vitreous humour, whereas for PEth and GGT in blood, there were highly significant trends between the biomarker levels and number of pathology classes. Although there were significant trends for EtG and EtS in urine, the main difference was between those without versus with any evidence of alcohol-related pathology. It is important to note that while the ethanol concentrations in blood and urine were available to the forensic expert shortly after autopsy, the results for the alcohol biomarkers were not available prior to determining the cause of death.

**Table 3:** Mean and median levels of biomarkers of alcohol or alcohol-induced damage by number of potentially alcohol-related classes of pathology

Biomarker	Statistics	Number of positive classes of pathology					Total
		0	1	2	3+	P-value*	
Ethanol in blood mg/g	Mean	1.19	1.55	1.35	1.07	0.376	1.37
	SD	1.29	1.61	1.69	1.44		1.55
	Median	0.73	1.36	0.41	0.00		0.74
	*n*	138	226	127	43		534
Ethanol in urine mg/g	Mean	1.57	2.11	1.84	1.54	0.860	1.86
	SD	1.61	1.91	1.99	1.84		1.86
	Median	1.15	2.47	0.98	0.00		1.63
	*n*	114	171	102	25		412
Ethanol in vitreous humour mg/g	Mean	1.02	1.83	1.81	1.54	0.332	1.60
	SD	1.45	1.87	1.95	1.92		1.81
	Median	0.00	2.05	1.49	0.00		0.61
	*n*	37	67	31	17		152
GGT in serum U/l	Mean	90.60	180.90	236.23	283.72	<0.001	182.02
	SD	84.02	259.44	302.79	250.83		248.01
	Median	55.00	89.00	128.50	197		92.00
	*n*	86	145	88	32		351
PEth in blood μmol/l	Mean	7.23	17.03	16.14	25.10	<0.001	15.23
	SD	9.68	20.75	15.41	23.21		18.30
	Median	3.06	9.72	12.14	17.95		9.75
	*n*	55	104	58	20		237
EtG in urine mg/l	Mean	105.00	377.52	331.61	349.79	0.033	288.13
	SD	225.82	559.16	593.89	398.19		500.71
	Median	12.70	130.50	82.75	216.30		70.50
	*n*	41	60	38	8		147
EtS in urine mg/l	Mean	24.01	75.72	62.88	56.99	0.043	56.96
	SD	52.18	102.71	103.73	62.67		91.56
	Median	3.70	35.35	23.10	40.40		20.00
	*n*	41	60	38	8		147

* *P*-value for trend in means of log biomarker values. Numbers of autopsies with biomarker concentrations varied by biomarker type as biomarkers could not be measured in all cases (material was haemolysed or putrefied). EtG = ethyl glucuronide; EtS = ethyl sulphate; GGT = gamma-glutamyl transferase; PEth = phosphatidylethanol; SD = standard deviation.

Table 4 shows the distribution of autopsies by number of alcohol-affected organs in relation to the frequency of drinking as reported by proxy informants (*n* = 144). After excluding the non-drinkers, there was a clear increasing trend in the percentage of cases with two or more pathology classes as drinking frequency increased (*P* = 0.010).

**Table 4:** Distribution of autopsies by proxy-reported frequency of alcohol drinking and number of positive classes of pathology

Frequency of drinking		Number of positive classes of pathology			
	0	1	2+	Total
Never or almost never	*n*	5	6	5	16
	%	31.3	37.5	31.3	100.0
3 times per month or less	*n*	8	22	11	41
	%	19.5	53.7	26.9	100.0
1–4 times per week	*n*	10	15	17	42
	%	23.8	35.7	40.5	100.0
Every day or almost every day	*n*	8	16	21	45
	%	17.8	35.6	46.7	100.0
Total	*n*	31	59	54	144
	%	20.5	41.0	37.5	100.0

This is based on the subset of subjects for whom proxy-derived information on frequency of drinking was available, and for whom information on presence or absence of each class of pathology was also recorded at autopsy.

Table 5 shows the frequencies of all pairwise combinations of potentially alcohol-related pathologies. Within each class of non-liver pathology, there was a high frequency of liver pathology, while only a minority of those with liver pathology showed a second type of pathology. It is notable that 70% of those with heart pathology also had liver pathology.

**Table 5:** Numbers and percentages^a^ of deaths with various pair-wise combinations of potentially alcohol-related classes of pathology

Organ-pathology	Liver (n *=* 335)	Pancreas (n *=* 78)	Lung (n *=* 103)	Stomach (n *=* 97)	Oesophagus (n *=* 8)	Heart (n *=* 30)
Liver	–	71/78 (91.0)	68/103 (66.0)	57/97 (58.8)	7/8 (87.5)	21/30 (70.0)
Pancreas	71/335 (21.2)	–	13/103 (12.6)	20/97 (20.6)	3/8 (37.5)	8/30 (26.7)
Lung	68/335 (20.3)	13/78 (16.7)	–	14/97 (14.4)	2/8 (25.0)	9/30 (30.0)
Stomach	57/335 (17.0)	20/78 (25.6)	14/103 (13.6)	–	1/8 (12.5)	5/30 (16.7)
Oesophagus	7/335 (2.1)	3/78 (3.8)	2/103 (1.9)	1/97 (1.0)	–	0/30 (0.0)
Heart	21/335 (6.5)	8/78(10.3)	9/103 (8.7)	5/97(5.2)	0/8 (0.0)	–

a Percentages of deaths with a defined (column) pathology with a second (row) pathology.

Table 6 shows the frequency of each pathology class according to the single underlying cause of death, as assigned in the death registry of the Estonian Statistical Office. As expected, liver pathology was found in all diagnoses of explicitly alcohol-related end-organ damage and of alcohol dependence, and in the vast majority of diagnoses of acute alcohol poisoning and diseases of the digestive system. For the causes of alcohol-related end-organ damage and of alcohol dependence, the frequency of pancreas pathology was also high (71 and 35%, respectively). There was a relatively high percentage of lung pathology (18%) in deaths from acute alcohol poisoning. Regarding deaths from diseases of the circulatory system, an intriguing finding was that more than two-thirds had evidence of potentially alcohol-related liver pathology. In deaths with an underlying cause of diseases of the digestive system, 92% had liver pathology and 58% pancreas pathology. With regard to deaths from respiratory causes, 77% had liver pathology. In external causes of death, liver and lung pathology were the most frequent, identified in ∼50% and ∼20% of cases.

**Table 6:** Frequencies (percentage^a^) of different potentially alcohol-related classes of pathology found at autopsy by underlying cause of death assigned in the Death Registry of the Estonian Statistical Office

Cause of death (ICD10 code)		Class of pathology						Deaths^b^
	Liver	Pancreas	Lung	Stomach	Oesophagus	Heart		
Explicitly alcohol related end-organ damage (I 42.6, K 70)	*n*	7	5	2	1	2	2	7
	%	100.0 (59.0–100.0^c^)	71.4 (29.0–96.3)	28.6 (3.7–71.0)	14.3 (0.4–57.9)	28.6 (3.7–71.0)	28.6 (3.7–70.9)	
	95% CI						
Alcohol dependence syndrome	*n*	34	12	4	7	0	4	34
(F10.2)	%	100.0 (89.7–100.0^c^)	35.3 (19.7–53.5)	11.8 (3.3–27.5)	20.6 (8.7–37.9)	0 (0.0–10.3^c^)	11.8 (3.3–27.5)	
	95% CI						
Acute alcohol poisoning (X45)	*n*	35	3	8	11	1	3	44
	%	79.5 (64.7–90.2)	6.8 (1.4–18.7)	18.2 (8.2–32.7)	25.0 (13.2–40.3)	2.3 (0.1–12.0)	6.8 (1.4–18.7)	
	95% CI						
External causes (V01–Y98, except X45)	*n*	160	33	65	59	1	10	330
	%	48.5 (43.0–54.0)	10.0 (7.0–13.8)	19.7 (15.5–24.4)	17.9 (14.0–22.4)	0.3 (0.0–1.7)	3.0 (1.5–5.5)	
	95% CI						
Diseases of the circulatory system (I00–I99, excluding I42)	*n*	61	12	6	7	1	1	88
	%	69.3 (58.6–78.7)	13.6 (7.2–22.6)	6.8 (2.5–14.3)	8.0 (3.3–15.7)	1.1 (0.0–6.2)	1.1 (0.0–6.2)	
	95% CI						
Other cardiomyopathies (I42 excluding I42.6)	*n*	3	1	1	2	0	3	4
	%	75.0 (19.4–99.4)	25.0 (0.6–80.6)	25.0 (0.6–80.6)	50.0 (6.8–93.2)	0 (0.0–60.2^c^)	75.0 (19.4–99.4)	
	95% CI						
Diseases of the digestive system (K00–K93, excluding K70)	*n*	11	7	2	3	2	2	44
	%	25.0 (13.2–40.3)	15.9 (6.6–30.1)	4.5 (0.6–15.5)	6.8 (1.4–18.7)	4.5 (0.6–15.5)	4.5 (0.6–15.5)	
	95% CI						
Diseases of the respiratory system (J00–J99)	*n*	10	1	13	2	1	1	13
	%	76.9 (46.2–95.0)	7.7 (0.2–36.0)	100.0 (75.3–100.0^c^)	15.4 (1.9–45.4)	7.7 (0.2–36.0)	7.7 (0.2–36.0)	
	95% CI						
All other causes	*n*	9	3	2	4	0	1	12
	%	75.0 (42.8–94.5)	25.0 (5.5–57.2)	16.7 (2.1–48.4)	33.3 (9.9–65.1)	0.0 (0.0–26.5^c^)	8.3 (0.2–38.5)	
	95% CI						

a Percentage of deaths in each underlying cause group with specified pathology.

b Number of deaths in each underlying cause group.

c One-sided, 97.5% confidence interval.

The age-adjusted odds ratio of dying from an alcohol-related cause versus from other causes was 4.8 [95% confidence interval (CI) = 1.8–12.5] for those with one positive pathology class, and 6.7 (95% CI = 2.6–17.7) for those with two or more classes, relative to those without positive classes of pathology.

## Discussion

In this study we have demonstrated that potentially alcohol-related pathologies are very common among working age men subject to forensic autopsy in Estonia, indicating high levels of habitual and harmful alcohol consumption in this group. Three-quarters of the cases showed evidence of alcohol-related pathology in one or more of the organs considered, with every third death having evidence of two or more pathologies. The liver was the organ with the highest prevalence of pathology (∼60%), with the pancreas, stomach and lung each showing evidence of pathology in about one in six cases. Only one in 20 showed evidence of changes to the heart that were identified as cardiomyopathy at post-mortem.

One of the most striking findings was that the majority of cases showing evidence of potentially alcohol-related pathology of the pancreas, lung, stomach, oesophagus or heart also showed evidence of liver damage. However, the reverse was far less common: among those with liver pathology, only a minority showed evidence of damage to other organs. This is consistent with alcohol-induced damage to other organs generally occurring only simultaneously or following damage to the liver. The associations observed between number of pathologies and biomarker levels and proxy measures of alcohol consumption are consistent with the risk of multi-organ damage being related to the ethanol dose, with the liver being the organ most likely to be affected first. There are several possible explanations for this. One is that the liver is especially vulnerable to alcohol-related damage, because it is exposed to high ethanol concentrations in the blood transported via the portal vein directly from the gut, with first-pass metabolism greatly lowering ethanol concentrations in the systemic circulation. However, this is not supported by animal studies showing that hepatic alcohol dehydrogenase is rapidly overwhelmed by ingested alcohol, and any first-pass mechanism is a consequence of alcohol dehydrogenase within the gastric mucosa 19. Another possible explanation is that alcohol-exposed hepatocytes are especially vulnerable to external stress, such as by proinflammatory cytokines produced by Kupffer cells 20. However, the precise reason why an individual heavy drinker does or does not experience damage to particular organs in addition to the liver is not yet fully understood.

### Problems with underlying cause of death

The presence of multiple alcohol-related pathologies at autopsy creates a problem for assigning a single underlying cause of death. The forensic pathologist may conclude that long-term heavy drinking is the causal factor. Nevertheless, current guidelines for assigning the underlying cause based on the 10th revision of the International Classification of Diseases (ICD-10) advise that use of the code for ‘mental and behavioural disorders due to alcohol’ (F10) should not be employed in the presence of specified types of end-organ damage; one of these pathologies should be listed instead as the underlying cause. In the presence of several pathologies, there is the further conundrum as to which one to choose. In Estonia, where alcohol-related damage to multiple organs is common, a broad forensic diagnosis of ‘chronic end-organ damage by alcohol’ is often used, rather than choosing in an arbitrary fashion any single organ pathology as the prime cause of death. In Estonia these deaths have been assigned by the Statistical Office as having the underlying cause of F10.2, because no single organ is mentioned. This explains the high prevalence of alcohol-related pathologies found among deaths where the underlying cause was coded to F10.2.

Our analyses have revealed a further complication when the immediate cause of death could plausibly be assigned to acute alcohol poisoning based on the blood ethanol concentration. We found an unexpectedly high proportion of deaths that had multiple pathologies detected certified as being due to this cause. This suggests that the majority of such poisoning deaths occurred among men who were regular heavy drinkers, rather than their death being the result of a one-off heavy drinking episode. Moreover, the presence of multiple pathologies may well have contributed to death. A recent study of sudden or unnatural deaths involving very high-level direct ethanol concentrations concluded similarly that these deaths were not due simply to the direct toxic effects of ethanol, but involved the consequences of a long history of alcohol problems 21. From the forensic pathologist's point of view, the reverse situation is also possible; when liver damage due to chronic alcohol consumption has occurred, lower blood ethanol concentrations can cause poisoning. In Estonia, it is formally recommended that a blood ethanol concentration ≥ 3.0 mg/g should be present, in order to assign acute alcohol poisoning as a cause of death 22. However, in their everyday practice the forensic pathologists, confronted with accompanying liver cirrhosis, may diagnose alcohol poisoning with a lower value, such as ≥ 2.5 mg/g. In this sense there may be an element of misclassification of acute alcohol-related poisonings acting in both directions.

For deaths from external causes (excluding acute poisoning), there is a moderately high prevalence of damage to one or more organs that could be attributed to alcohol. Most of these deaths are not the consequence of isolated episodes of drunkenness. Rather, they may be occurring disproportionately among men with a pattern of alcohol consumption that gave rise to organic pathologies as well as putting them at risk of deaths from external causes.

### Generalizability

We have examined the identification of multiple alcohol-related pathologies in a very particular group of deaths that were subject to forensic autopsy in Estonia. In this period, forensic autopsies were conducted only for a subset of deaths where the circumstances or nature of the death required formal investigation, such as poisonings, injury or sudden and unexpected deaths. To this extent, the deaths we studied cannot be considered as representative of all deaths in the target age group. However, as our results confirm, these deaths included a high proportion of heavy drinkers, as evidenced by the large proportion found with liver damage and also reported to be regular alcohol drinkers. In this respect they provide an ideal group in which to investigate the coexistence of multiple alcohol-related pathologies. The fact that the deaths studied are not representative of any set of deaths except those subject to forensic autopsy does not matter. The challenge of assigning a single underlying cause of death remains the same for the members of this group who had multiple alcohol-related pathologies as it would be for any death related to alcohol, even where no autopsy was performed. Of course, the limitation of studying forensic autopsies is that we cannot use our results to draw inferences about the prevalence of alcohol-related pathologies in all deaths occurring in Estonia. However, this was not an objective of the study.

Although these findings originate from only one country, similar findings are also likely to be obtained in other settings where there are subgroups in the population who drink heavily. The present observations are therefore likely to have a more general significance for death certification. Heavy alcohol consumption causes damage to a wide range of organs, yet the death registration lists only a single underlying cause. Given other evidence of international variation in cause-of-death coding 23, even when the cause is seemingly obvious 24, this may also hold true for alcohol-related causes. Accordingly this may produce artefactual variations in death rates from single causes such as liver cirrhosis, which is widely used as the cardinal indicator of alcohol-related disease in a population. Countries with a high prevalence of spirit drinking, for example, where heavy drinkers are at a particularly high risk of death from acute alcohol poisoning or fatal intoxication due to inhalation of vomit, may have rates of fatal or life-threatening liver cirrhosis that are far higher than is apparent from mortality rates from cirrhosis *per se*. This raises a broader question of whether there is a need for a new conceptualization of the cause of death that captures the diverse range of alcohol-related pathologies.

### Alcohol biomarkers

Some alcohol biomarkers were found to be associated with the number of potentially alcohol-related pathologies: PEth and GGT in blood, and EtG and EtS in urine. In contrast, the ethanol concentrations in blood, urine and vitreous humour were not related to the number of pathologies. Thus, although at autopsy the ethanol concentration is used commonly to determine whether acute intoxication is implicated as a cause of death, it is not informative about whether a death might be attributable to long-term harmful drinking. We have shown previously that at post-mortem GGT and EtG were the markers related most strongly to proxy-reported frequency of drinking 16. Recently Rainio *et al*. have shown that measuring vitreous humour EtG by immunoassay is a useful forensic tool for screening for ante-mortem alcohol use 25. In a cross-sectional study of live men 26, GGT was found to be the second best alcohol biomarker after carbohydrate-deficient transferrin (CDT), with high specificity for current drinking. However, in post-mortem analysis the high prevalence of haemolysis of blood samples makes CDT a less useful test 16. For this reason, measurement of CDT in vitreous humour is probably more valuable 27.

### Limitations

In Estonia today only a minority of deaths, even at working ages (0.15%; 0.25% for men and 0.05% for women), are subject to forensic autopsy, and these will differ in many respects from other deaths. The circumstances in which a forensic autopsy is performed in Estonia have been described previously 16. According to data from the Estonian Forensic Science Institute, about 90% of forensic autopsy cases are tested for alcohol and 35–40% of these have a blood ethanol concentration exceeding 0.5 mg/g. However, in another respect the high prevalence of heavy drinkers among deaths subjected to forensic autopsy provides an excellent source of cases for understanding more about the presence of multiple alcohol-related pathologies.

### Implications for policy

Our findings are timely, as the eleventh edition of the ICD is now being prepared. The World Health Organization encourages broad participation in the 11th revision so that the final classification meets the needs of health information users and is more comprehensive 28.

### Declaration of interests

None.
